# Bioremediation techniques–classification based on site of application: principles, advantages, limitations and prospects

**DOI:** 10.1007/s11274-016-2137-x

**Published:** 2016-09-16

**Authors:** Christopher Chibueze Azubuike, Chioma Blaise Chikere, Gideon Chijioke Okpokwasili

**Affiliations:** Department of Microbiology, Faculty of Science, University of Port Harcourt, East-West Road, PMB 5323, Choba, Port Harcourt, 500004 Rivers State Nigeria

**Keywords:** Bioremediation, Environment, Pollutants, Techniques

## Abstract

Environmental pollution has been on the rise in the past few decades owing to increased human activities on energy reservoirs, unsafe agricultural practices and rapid industrialization. Amongst the pollutants that are of environmental and public health concerns due to their toxicities are: heavy metals, nuclear wastes, pesticides, green house gases, and hydrocarbons. Remediation of polluted sites using microbial process (bioremediation) has proven effective and reliable due to its eco-friendly features. Bioremediation can either be carried out ex situ or in situ, depending on several factors, which include but not limited to cost, site characteristics, type and concentration of pollutants. Generally, ex situ techniques apparently are more expensive compared to in situ techniques as a result of additional cost attributable to excavation. However, cost of on-site installation of equipment, and inability to effectively visualize and control the subsurface of polluted sites are of major concerns when carrying out in situ bioremediation. Therefore, choosing appropriate bioremediation technique, which will effectively reduce pollutant concentrations to an innocuous state, is crucial for a successful bioremediation project. Furthermore, the two major approaches to enhance bioremediation are biostimulation and bioaugmentation provided that environmental factors, which determine the success of bioremediation, are maintained at optimal range. This review provides more insight into the two major bioremediation techniques, their principles, advantages, limitations and prospects.

## Introduction

**Fig. 1:**
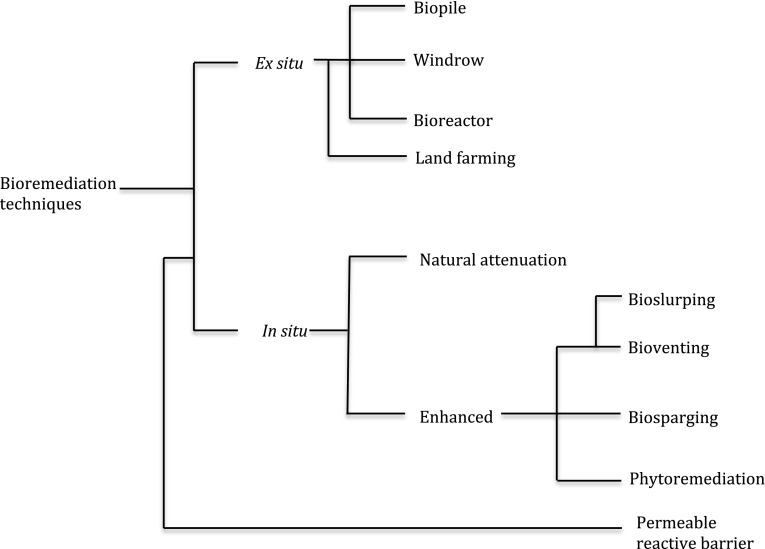
Bioremediation techniques. The divergence of each technique is hypothetical; therefore, the *left to right* order of internal nodes are not the order of evolution (technique development). Permeable reactive barrier (PBR) is not the arbitrary tree root. It is a physical remediation technique with some elements of bioremediation, hence the early hypothetical divergence

In the past two decades, there have been recent advances in bioremediation techniques with the ultimate goal being to effectively restore polluted environments in an eco-friendly approach, and at a very low cost. Researchers have developed and modelled different bioremediation techniques; however, due to nature and/or type of pollutant, there is no single bioremediation technique that serves as a ‘*silver bullet’* to restore polluted environments. Autochthonous (indigenous) microorganisms present in polluted environments hold the key to solving most of the challenges associated with biodegradation and bioremediation of polluting substances (Verma and Jaiswal 2016) provided that environmental conditions are suitable for their growth and metabolism. Environmentally friendly and cost saving features are amongst the major advantages of bioremediation compared to both chemical and physical methods of remediation. Thus far, several good definitions have been given to bioremediation, with particular emphasis on one of the processes (degradation). Nevertheless, in some instances, the term biodegradation is used interchangeably with bioremediation; the former is a term, which applies to a process under the latter. In this review, bioremediation is defined as a process, which relies on biological mechanisms to reduce (degrade, detoxify, mineralize or transform) concentration of pollutants to an innocuous state. The process of pollutant removal depends primarily on the nature of the pollutant, which may include: agrochemicals, chlorinated compounds, dyes, greenhouse gases, heavy metals, hydrocarbons, nuclear waste, plastics, and sewage. Apparently, taking into consideration site of application, bioremediation techniques can be categorized as: ex situ or in situ. Pollutant nature, depth and degree of pollution, type of environment, location, cost, and environmental policies are some of the selection criteria that are considered when choosing any bioremediation technique (Frutos et al. 2012; Smith et al. 2015). Apart from selection criteria, performance criteria (oxygen and nutrient concentrations, temperature, pH, and other abiotic factors) that determine the success of bioremediation processes are also given major considerations prior to bioremediation project. Although bioremediation techniques are diverse (Fig. 1), most studies on bioremediation are focused on hydrocarbons on account of frequent pollution of soil and ground water with this particular type of pollutant (Frutos et al. 2010; Sui and Li 2011; Kim et al. 2014; Firmino et al. 2015). Besides, it is possible that other remediation techniques (Pavel and Gavrilescu 2008), which might as well be more economical, and efficient to apply during remediation, are considered when remediation of sites polluted with pollutants aside from hydrocarbons are involved. Furthermore, given the nature of activities leading to crude oil pollution, it is likely that pollution of the environment with pollutants excluding hydrocarbons can easily be prevented and controlled. Moreover, the dependence on petroleum and other related products as major sources of energy seems to have contributed to increased pollution resulting from this class of pollutant (Gomez and Sartaj 2013; Khudur et al. 2015). The aim of this review is to provide a comprehensive knowledge on the two major bioremediation techniques with regards to site of application, highlighting their principles, advantages, limitations and possible solutions. The prospects of bioremediation are also discussed.

## Ex situ bioremediation techniques

These techniques involve excavating pollutants from polluted sites and subsequently transporting them to another site for treatment. Ex situ bioremediation techniques are usually considered based on: the cost of treatment, depth of pollution, type of pollutant, degree of pollution, geographical location and geology of the polluted site. Performance criteria, which also determine the choice of ex situ bioremediation techniques, have been described (Philp and Atlas 2005).

### Biopile

Biopile-mediated bioremediation involves above-ground piling of excavated polluted soil, followed by nutrient amendment, and sometimes aeration to enhance bioremediation by basically increasing microbial activities. The components of this technique are: aeration, irrigation, nutrient and leachate collection systems, and a treatment bed. The use of this particular ex situ technique is increasingly being considered due to its constructive features including cost effectiveness, which enables effective biodegradation on the condition that nutrient, temperature and aeration are adequately controlled (Whelan et al. 2015). The application of biopile to polluted sites can help limit volatilization of low molecular weight (LMW) pollutants; it can also be used effectively to remediate polluted extreme environments such as the very cold regions (Dias et al. 2015; Gomez and Sartaj 2014; Whelan et al. 2015). In line with this, Gomez and Sartaj (2014) studied the effects of different application rates (3 and 6 ml/m^3^) of microbial consortia, and mature compost (5 and 10 %) on total petroleum hydrocarbon (TPH) reduction in field-scale biopiles at low temperature conditions, using response surface methodology (RSM) based on factorial design of experiment (DoE) tone. At the end of the study period (94 days), 90.7 % TPH reduction in the bioaugmented and biostimulated setups were obtained compared to the control setups with 48% average TPH removal. The high percentage of TPH reduction was attributed to synergistic interaction between bioaugmentation and biostimulation, thus demonstrating the flexibility of biopiles for bioremediation. Similarly, Dias et al. (2015) reported 71 % reduction in total hydrocarbon concentration, and a shift in bacterial structure over 50-day study period following pretreatment of contaminated soil samples prior to biopile formation, and subsequent biostimulation with fishmeal. The feasibility of biopiles towards bioremediation of different soil samples including clay and sandy soil has been reported (Chemlal et al. 2013; Akbari and Ghoshal 2014). The flexibility of biopile allows remediation time to be shortened as heating system can be incorporated into biopile design to increase microbial activities and contaminant availability thus increasing the rate of biodegradation (Aislabie et al. 2006). Furthermore, heated air can be injected into biopile design to deliver air and heat in tandem, in order to facilitate enhanced bioremediation. In another study, Sanscartier et al. (2009) reported that humidified biopile had a very low final TPH concentration compared to heated and passive biopiles as a result of optimal moisture content, reduced leaching, minimal volatilization of less degradable contaminants. In addition, it was reported that biopile could be used to treat large volume of polluted soil in a limited space. Biopile setup can easily be scaled up to a pilot system to achieve similar performance obtained during laboratory studies (Chemlal et al. 2013). Important to the efficiency of biopile is sieving and aeration of contaminated soil prior to processing (Delille et al. 2008). Bulking agents such as straw, saw dust, bark or wood chips and other organic materials have been added to enhance remediation process in a biopile construct (Rodríguez-Rodríguez et al. 2010).

Although biopile systems conserve space compared to other field ex situ bioremediation techniques, including land farming, robust engineering, cost of maintenance and operation, lack of power supply especially at remote sites, which would enable uniform distribution of air in contaminated piled soil via air pump are some of the limitations of biopiles. More so, excessive heating of air can lead to drying of soil undergoing bioremediation, which will result in inhibition of microbial activities, and promote volatilization rather than biodegradation (Sanscartier et al. 2009).

### Windrows

As one of ex situ bioremediation techniques, windrows rely on periodic turning of piled polluted soil to enhance bioremediation by increasing degradation activities of indigenous and/or transient hydrocarbonoclastic bacteria present in polluted soil. The periodic turning of polluted soil, together with addition of water bring about increase in aeration, uniform distribution of pollutants, nutrients and microbial degradative activities, thus speeding up the rate of bioremediation, which can be accomplished through assimilation, biotransformation and mineralization (Barr 2002). Windrow treatment when compared to biopile treatment, showed higher rate of hydrocarbon removal; however, the higher efficiency of the windrow towards hydrocarbon removal was as a result of the soil type, which was reported to be more friable (Coulon et al. 2010). Nevertheless, due to periodic turning associated with windrow treatment, it may not be the best option to adopt in remediating soil polluted with toxic volatiles. The use of windrow treatment has been implicated in CH_4_ (greenhouse gas) release due to development of anaerobic zone within piled polluted soil, which usually occurs following reduced aeration (Hobson et al. 2005).

### Bioreactor

Bioreactor, as the name implies, is a vessel in which raw materials are converted to specific product(s) following series of biological reactions. There are different operating modes of bioreactor, which include: batch, fed-batch, sequencing batch, continuous and multistage. The choice of operating mode depends mostly on market economy and capital expenditure. Conditions in a bioreactor support natural process of cells by mimicking and maintaining their natural environment to provide optimum growth conditions. Polluted samples can be fed into a bioreactor either as dry matter or slurry; in either case, the use of bioreactor in treating polluted soil has several advantages compared to other ex situ bioremediation techniques. Excellent control of bioprocess parameters (temperature, pH, agitation and aeration rates, substrate and inoculum concentrations) is one of the major advantages of bioreactor-based bioremediation. The ability to control and manipulate process parameters in a bioreactor implies that biological reactions within can be enhanced to effectively reduce bioremediation time. Importantly, controlled bioaugmentation, nutrient addition, increased pollutant bioavailability, and mass transfer (contact between pollutant and microbes), which are among the limiting factors of bioremediation process can effectively be established in a bioreactor thus making bioreactor-based bioremediation more efficient. Further, it can be used to treat soil or water polluted with volatile organic compounds (VOCs) including benzene, toluene, ethylbenzene and xylenes (BTEX). The applications of different bioreactors for bioremediation process have resulted in removal of wide range of pollutants (Table 1). The flexible nature of bioreactor designs allows maximum biological degradation while minimizing abiotic losses (Mohan et al. 2004). Short or long-term operation of a bioreactor containing crude oil-polluted soil slurry allows tracking of changes in microbial population dynamics thus enabling easy characterization of core bacterial communities involved in bioremediation processes (Chikere et al. 2012; Zangi-Kotler et al. 2015). Furthermore, it allows the use of different substances as biostimulant or bioaugmenting agent including sewage sludge. In addition, bioreactor being an enclosed system, genetically modified microorganism (GEM) can be used for bioaugmentation after which the organism (GEM) can be destroyed before treated soils are returned to field for landfilling. This containment of GEM in a bioreactor followed by destruction will help ensure that no foreign gene escapes into an environment after bioremediation. With bioreactor, the role of biosurfactant was found to be insignificant due to efficient mixing associated with bioreactor operations (Mustafa et al. 2015).

**Table 1:** Some pollutants removed by bioreactor-based bioremediation

Type/mode of bioreactor operation	Nature of sample	Nature of pollutant	Initial concentration	% Removal	References
Stir tank bioreactor (2.5 L)	Crude oil polluted sediment	Total petroleum and polyaromatic hydrocarbons	19 and 3.1 ppm respectively	82–97	Chikere et al. (2016)
Stir tank bioreactor/batch (1.5 L)	Waste lubricating oil	Saturated and aromatic hydrocarbons	80–86 g/L	62–69	Bhattacharya et al. (2015)
Expanded granular sludge bed (EGSB) reactor (1.4 L)	Laundry wastewater	Linear alkylbenze sulfonate (LAS)	7.0 g TVS/L	92.9	Delforno et al. (2015)
Anaerobic sludge blanket/continuous-flow (3.3 L)	Synthetic BTEX-contaminated water	Benzene, toluene, ethylbeneze, and xylene (BTEX)	50 g VSS/L	51–86	Firmino et al. (2015)
Packed-bed reactor (PBR, 1.25 L)	Amines	Mixture of sulfonated amines (4-aminobenzene sulfonic acid and 4-amino naphthalene sulfonic acid)	50 mg/L		Juárez-Ramírez et al. (2015)
Roller slurry bioreactor (1 L)	Contaminated soil	2,4-Dichlorophenoxyacetic Acid	200–500 mg/kg	97–100	Mustafa et al. (2015)
Packed bed biofilter (100 cm × 5 cm)	Pharmaceutical sludge	Xylene vapour	0.2–1.2 g/m^3^	95–99	Saravanan et al. (2015)
Submerged attached growth bioreactors (SAGBs, 61 cm × 61 cm × 46 cm)	Effluent	Total nitrogen		48–53	Shannon et al. (2015)^a^
Membrane bioreactor (MBR, 8 L)	Coal gasification wastewater	Naphthalene and total nitrogen	10–200 mg/L	48–98	Xu et al. (2015)
Sequencing batch reactors (SBR, 2.5 L)	Engineered nanomaterials (ENMs)	Nano fullerenes (nC60) and nanosilver		>90 %	Yang et al. (2015)
Miniature membrane bioreactor (mMBR)/continuous	Brominated flame retardants (BFR)	Dibromoeopentyl glycol (DBNPG)		50	Zangi-Kotler et al. (2015)
Sequence batch reactor (1.5 L)	Contaminated soil	Carbofuran	20 mg/kg	88–97	Plangklang and Alissara Reungsang (2010)
Glass jar paddle-type impeller reactor (2 L)	Contaminated soil	2,4,6-trinitophenylmethylnitramine (tetryl)	1,00,000 mg/kg	99.9	Fuller et al. (2003)

^a^Pilot study

Despite that bioreactor-based bioremediation has proven to be efficient as a result of different operating parameters, which can easily be controlled, establishing best operating condition by relating all parameters using one-factor-at-a-time (OFAT) approach would likely require numerous experiments, which is time-consuming. This particular challenge can be overcome by using design of experiment (DoE) tone, which provides information on optimal range of parameters using a set of independent variables (controllable and uncontrollable factors) over a specified region (level) (Mohan et al. 2007). Notwithstanding, understanding microbiological processes is of great importance when optimizing bioremediation processes (Piskonen et al. 2005). Moreover, bioreactor-based bioremediation is not a popular full-scale practice due to some reasons. Firstly, due to bioreactor being ex situ technique, the volume of polluted soil or other substances to be treated may be too large, requiring more manpower, capital and safety measures for transporting pollutant to treatment site, therefore, making this particular technique cost ineffective (Philp and Atlas 2005). Secondly, due to several bioprocess parameters or variables of a bioreactor, any parameter that is not properly controlled and/or maintained at optimum, may become a limiting factor; this in turn will reduce microbial activities and will make bioreactor-based bioremediation process less effective. Lastly, pollutants are likely to respond differently to different bioreactors; the availability of the most suitable design is of paramount importance. Above all, cost of a bioreactor suitable for a laboratory or pilot-scale bioremediation makes this technique to be capitally intensive.

### Land farming

Land farming is amongst the simplest bioremediation techniques owing to its low cost and less equipment requirement for operation. In most cases, it is regarded as ex situ bioremediation, while in some cases, it is regarded as in situ bioremediation technique. This debate is due to the site of treatment. Pollutant depth plays an important role as to whether land farming can be carried out ex situ or in situ. In land farming, one thing is common, polluted soils are usually excavated and/or tilled, but the site of treatment apparently determines the type of bioremediation. When excavated polluted soil is treated on-site, it can be regarded as in situ; otherwise, it is ex situ as it has more in common with other ex situ bioremediation techniques. It has been reported that when a pollutant lies <1 m below ground surface, bioremediation might proceed without excavation, while pollutant lying >1.7 m needs to be transported to ground surface for bioremediation to be effectively enhanced (Nikolopoulou et al. 2013). Generally, excavated polluted soils are carefully applied on a fixed layer support above the ground surface to allow aerobic biodegradation of pollutant by autochthonous microorganisms (Philp and Atlas 2005; Paudyn et al. 2008; Volpe et al. 2012; Silva-Castro et al. 2015). Tillage, which brings about aeration, addition of nutrients (nitrogen, phosphorus and potassium) and irrigation are the major operations, which stimulate activities of autochthonous microorganisms to enhance bioremediation during land farming. Nevertheless, it was reported that tillage and irrigation without nutrient addition in a soil with appropriate biological activity increased heterotrophic and diesel-degrading bacterial counts thus enhancing the rate of bioremediation; dehydrogenase activity was also observed to be a good indicator of biostimulation treatment and could be used as a biological parameter in land farming technology (Silva-Castro et al. 2015). Similarly, in a field trial, Paudyn et al. (2008) reported >80 % contaminant (diesel) removal by aeration using rototilling approach at remote Canadian Arctic location over a 3-year study period; this further demonstrates that in land farming technique, aeration plays crucial role in pollutant removal especially at cold regions. Land farming is usually used for remediation of hydrocarbon-polluted sites including polyaromatic hydrocarbons (Silva-Castro et al. 2012; Cerqueira et al. 2014); as a result, biodegradation and volatilization (weathering) are the two remediation mechanisms involved in pollutant removal. Land farming system complies with government regulations, and can be used in any climate and location (Besaltatpour et al. 2011). The construction of a suitable land farming design with an impermeable liner minimizes leaching of pollutant into neighbouring areas during bioremediation operation (da Silva et al. 2012). Over all, land farming bioremediation technique is very simple to design and implement, requires low capital input and can be used to treat large volume of polluted soil with minimal environmental impact and energy requirement (Maila and Colete 2004).

Although the simplest bioremediation technique, land farming like other ex situ bioremediation techniques has some limitations, which include: large operating space, reduction in microbial activities due to unfavourable environmental conditions, additional cost due to excavation, and reduced efficacy in inorganic pollutant removal (Khan et al. 2004; Maila and Colete 2004). Moreover, it is not suitable for treating soil polluted with toxic volatiles due to its design and mechanism of pollutant removal (volatilization), especially in hot (tropical) climate regions. These limitations and several others make land farming-based bioremediation time consuming and less efficient compared to other ex situ bioremediation techniques.

One of the major advantages of ex situ bioremediation techniques is that they do not require extensive preliminary assessment of polluted site prior to remediation; this makes the preliminary stage short, less laborious and less expensive. Due to excavation processes associated with ex situ bioremediation, pollutant inhomogeneity as a result of depth, non-uniform concentration and distribution, can easily be curbed by effectively optimizing some process parameters (temperature, pH, mixing) of any ex situ technique to enhance bioremediation process. These techniques allow modifications of biological, chemical and physico-chemical conditions and parameters necessary for effective and efficient bioremediation. Importantly, the great influence of soil porosity, which governs transport processes during remediation, can be reduced when polluted soils are excavated. Ex situ bioremediation techniques are unlikely to be used in some sites such as under buildings, inner city and working sites (Philp and Atlas 2005). On the other hand, the excavation features of ex situ bioremediation tend to disrupt soil structure; as a result, polluted and surrounding sites alike experience more disturbances. Moderate to extensive engineering required for any ex situ bioremediation techniques implies that more workforce and capital are required to construct any of the technique. In most cases, these techniques require large space for operation. Generally, ex situ bioremediation techniques tend to be faster, easier to control and can be used to treat wide range of pollutants (Prokop et al. 2000).

## In situ bioremediation techniques

These techniques involve treating polluted substances at the site of pollution. It does not require any excavation; therefore, it is accompanied by little or no disturbance to soil structure. Ideally, these techniques ought to be less expensive compared to ex situ bioremediation techniques, due to no extra cost required for excavation processes; nonetheless, cost of design and on-site installation of some sophisticated equipment to improve microbial activities during bioremediation is of major concern. Some in situ bioremediation techniques might be enhanced (bioventing, biosparging and phytoremediation), while others might proceed without any form of enhancement (intrinsic bioremediation or natural attenuation). In situ bioremediation techniques have been successfully used to treat chlorinated solvents, dyes, heavy metals, and hydrocarbons polluted sites (Folch et al. 2013; Kim et al. 2014; Frascari et al. 2015; Roy et al. 2015). Notably, the status of electron acceptor, moisture content, nutrient availability, pH and temperature are amongst the important environmental conditions that need to be suitable for a successful in situ bioremediation to be achieved (Philp and Atlas 2005). Unlike ex situ bioremediation techniques, soil porosity strongly influences the application of in situ bioremediation to any polluted site.

## Enhanced in situ bioremediation

### Bioventing

This technique involve controlled stimulation of airflow by delivering oxygen to unsaturated (vadose) zone in order to increase bioremediation, by increasing activities of indigenous microbes. In bioventing, amendments are made by adding nutrients and moisture to enhance bioremediation with the ultimate goal being to achieve microbial transformation of pollutants to a harmless state (Philp and Atlas 2005). This technique has gained popularity among other in situ bioremediation techniques especially in restoring sites polluted with light spilled petroleum products (Höhener and Ponsin 2014). A study by Sui and Li (2011) modelled the effect of air injection rate on volatilization, biodegradation and biotransformation of toluene-contaminated site by bioventing. It was observed that at two different air injection rates (81.504 and 407.52 m^3^/d), no significant difference in contaminant (toluene) removal was observed at the end of the study period (200 days). However, at the earlier stage of the study (day 100), it was observed that high air injection rate resulted in enhanced toluene removal by volatilization compared to low air injection rate. In other words, high airflow rate does not bring about increase in biodegradation rate nor make pollutant biotransformation more effective. This is due to early saturation of air (by high or low air injection rate) in the subsurface for oxygen demand during biodegradation. Nonetheless, low air injection rate resulted in a significant increase in biodegradation. It thus demonstrates that in bioventing, air injection rate is among the basic parameters for pollutant dispersal, redistribution and surface loss. Similarly, Frutos et al. (2010) reported the effectiveness of bioventing treatment in remediation of phenanthrene-contaminated soil and recorded >93 % contaminant removal after 7 months. Airflow intensities and airflow intervals resulted in no significant difference in diesel removal from clayey soil, implying that longer air injection interval and low air injection rate might be more economical for bioventing in diesel-polluted clayey soil (Thomé et al. 2014). Interestingly, Rayner et al. (2007) observed that in a sub-Antarctic hydrocarbon-polluted site, single-well bioventing was ineffective towards hydrocarbon removal ascribable to shallow water table and thin soil cover, which led to channel development; whereas, when a microbioventing using nine small injection rods (0.5 m apart) was carried out on the same site, under identical conditions, a considerable amount of hydrocarbons were removed due to more uniform distribution of oxygen thus resulting in increased biodegradation. It becomes apparent that though airflow rates and air intervals are amongst the basic parameters of bioventing, the success of bioventing-based bioremediation relies on the number of air injection points, which helps to achieve uniform distribution of air. Despite the fact that bioventing design is to encourage aeration in unsaturated zone, it can be used for anaerobic bioremediation process especially in treating vadose zone polluted with chlorinated compounds, which are recalcitrant under aerobic conditions. In this latter process, in lieu of air or pure oxygen, mixture of nitrogen together with low concentrations of carbon dioxide and hydrogen can also be injected to bring about reduction of chlorinated vapour, with hydrogen acting as electron donor (Mihopoulos et al. 2000, 2002; Shah et al. 2001). In a soil with low-permeability, injection of pure oxygen might lead to higher oxygen concentration compared to air injection. Furthermore, ozonation might be useful for partial oxidation of recalcitrant compounds in order to accelerate biodegradation (Philp and Atlas 2005).

Unlike bioventing that relies on enhancing microbial degradation process at the vadose zone by moderate air injection, soil vapour extraction (SVE) maximizes volatile organic compound volatilization via vapour extraction (Magalhães et al. 2009). Although both techniques use identical hardware, the configuration, philosophical design and operation differ significantly (Diele et al. 2002). Airflow rate in SVE is higher compared to that of bioventing (Baker and Moore 2000). SVE may be regarded as physical method of remediation due to its mechanism of pollutant removal, however, the mechanism involved in pollutant removal for both techniques are not mutually exclusive.

During on-site field trials, achieving similar results obtained during laboratory studies is not always attainable due to other environmental factors and different characteristics of the unsaturated zone to which air is injected; as a result, with bioventing, treatment time may be prolonged. Apparently, high airflow rate leads to transfer of volatile organic compounds to the soil vapour phase, which requires off-gas treatment of the resulting gases prior to release into the atmosphere (Burgess et al. 2001). This particular challenge can be resolved by combining bioventing and biotrickling filter techniques to reduce both contaminant and outlet gas emission levels; thus reducing the extended treatment time associated with bioventing alone (Magalhães et al. 2009).

### Bioslurping

This technique combines vacuum-enhanced pumping, soil vapour extraction and bioventing to achieve soil and groundwater remediation by indirect provision of oxygen and stimulation of contaminant biodegradation (Gidarakos and Aivalioti 2007). The technique is designed for free products recovery such as light non-aqueous phase liquids (LNAPLs), thus remediating capillary, unsaturated and saturated zones. It can also be used to remediate soils contaminated with volatile and semi-volatile organic compounds. The system uses a “slurp” that extends into the free product layer, which draws up liquids (free products and soil gas) from this layer in a manner similar to that of how a straw draws liquid from any vessel. The pumping mechanism brings about upward movement of LNAPLs to the surface, where it becomes separated from water and air. Following complete free products removal, the system can easily be made to operate as a conventional bioventing system to complete remediation process (Kim et al. 2014). In this technique, excessive soil moisture limits air permeability and decreases oxygen transfer rate, in turn reducing microbial activities. Although the technique is not suitable for remediating soil with low permeability, it saves cost due to less amount of groundwater resulting from the operation thus minimizes storage, treatment and disposal costs (Philp and Atlas 2005). Establishing a vacuum on a deep high permeable site and fluctuating water table, which could create saturated soil lenses that are difficult to aerate are amongst the major concerns of this particular in situ technique.

### Biosparging

This technique is very similar to bioventing in that air is injected into soil subsurface to stimulate microbial activities in order to promote pollutant removal from polluted sites. However, unlike bioventing, air is injected at the saturated zone, which can cause upward movement of volatile organic compounds to the unsaturated zone to promote biodegradation. The effectiveness of biosparging depends on two major factors namely: soil permeability, which determines pollutant bioavailability to microorganisms, and pollutant biodegradability (Philp and Atlas 2005). As with bioventing and soil vapour extraction (SVE), biosparing is similar in operation with a closely related technique known as in situ air sparging (IAS), which relies on high airflow rates to achieve pollutant volatilization, whereas biosparging promotes biodegradation. Similarly, both mechanisms of pollutant removal are not mutually exclusive for both techniques. Biosparging has been widely used in treating aquifers contaminated with petroleum products, especially diesel and kerosene. Kao et al. (2008) reported that biosparging of benzene, toluene, ethylbenzene and xylene (BTEX)-contaminated aquifer plume resulted in a shift from anaerobic to aerobic conditions; this was evidenced by increased dissolved oxygen, redox potentials, nitrate, sulphate and total culturable heterotrophs with a corresponding decrease in dissolved ferrous iron, sulphide, methane and total anaerobes and methanogens. The over all decrease in BTEX reduction (>70 %) further indicates that biosparging can be used to remediate BTEX contaminated ground water. The major limitation however, is predicting the direction of airflow.

### Phytoremediation

This technique relies on the use of plant interactions (physical, biochemical, biological, chemical and microbiological) in polluted sites to mitigate the toxic effects of pollutants. Depending on pollutant type (elemental or organic), there are several mechanisms (accumulation or extraction, degradation, filtration, stabilization and volatilization) involved in phytoremediation. Elemental pollutants (toxic heavy metals and radionuclides) are mostly removed by extraction, transformation and sequesteration. On the other hand, organic pollutants (hydrocarbons and chlorinated compounds) are predominantly removed by degradation, rhizoremediation, stabilization and volatilization, with mineralization being possible when some plants such as willow and alfalfa are used (Meagher 2000; Kuiper et al. 2004). Some important factors to consider when choosing a plant as a phytoremediator include: root system, which may be fibrous or tap depending on the depth of pollutant, above ground biomass, which should not be available for animal consumption, toxicity of pollutant to plant, plant survival and its adaptability to prevailing environmental conditions, plant growth rate, site monitoring and above all, time required to achieve the desired level of cleanliness. In addition, the plant should be resistant to diseases and pests (Lee 2013). It has been reported (Miguel et al. 2013) that in some contaminated environments, the process of contaminant removal by plant involves: uptake, which is largely by passive process, translocation from roots to shoots, which is carried out by xylem flow, and accumulation in shoot. Further, translocation and accumulation depend on transpiration, and partitioning between xylem sap and adjacent tissues, respectively. Nonetheless, the process is likely to differ, depending on other factors such as nature of contaminant and plant type. It is plausible that most plants growing in any polluted site are good phytoremediators. Therefore, the success of any phytoremediation approach primarily depends on optimizing the remediation potentials of native plants growing in polluted sites either by bioaugmentation with endogenous or exogenous plant rhizobacteria, or by biostimulation. It was reported that the use of plant growth-promoting rhizobacteria (PGPR) might play an important role in phytoremediation, as PGPR tends to enhance biomass production and tolerance of plants to heavy metals and other unfavourable soil (edaphic) conditions (Yancheshmeh et al. 2011; de-Bashan et al. 2012). In addition, Grobelak et al. (2015) reported increased plant length, root and stem growth, when *Brassica napus* L. subsp. *napus* and *Festuca ovinia* L. were inoculated with exogenous PGPR during seed germination, and 2 weeks after plant growth; thus protecting the seeds and plants from growth inhibition on heavy metal-polluted soil. Similarly, during phytoremediation of metal-contaminated estuaries with *Spartina maritima*, bioaugmentation with endogenous rhizobacteria resulted in increased plant subsurface biomass, metal accumulation and enhanced metal removal (Mesa et al. 2015). Addition of biosurfactant produced by *Serratia marcescens* to gasoline-contaminated soil to which *Ludwigia octovalvis* were planted, resulted in 93.5 % total petroleum hydrocarbon (TPH) removal and corresponding increase in microbial count; this was attributed to desorption and solubilization effects of biosurfactant, which in turn increased gasoline bioavailability to microbial consortia within *L. octovalvis* rhizosphere (Almansoory et al. 2015). On the contrary, Maqbool et al. (2012) reported higher and rapid total petroleum hydrocarbon (TPH) removal in the rhizosphere of *Sesbania cannabina* uninoculated soil compared to that of inoculated soil. This was ascribed to the long fibrous root of the plant, which aided in proliferation of rhizobacteria and increased interaction with the contaminant, resulting in unfavourable competition in the rhizosphere of inoculated plant. Different plant species have been reported to have innate ability to remove organic and elemental pollutants from polluted sites (Table 2). *Brachiaria mutica* and *Zea mays* have also been reported as potential phytoremediators of heavy metal-contaminated sites (Ijaz et al. 2015; Tiecher et al. 2016). Other plants with phytoremediation potentials have been extensively described (Kuiper et al. 2004; Wang et al. 2012a, b; Ali et al. 2013; Yavari et al. 2015) and some transgenic plants for enhanced phytoremediation including genes transferred have also been described (Lee 2013).

**Table 2:** Some plants with phytoremediation potentials

Plant	Nature of pollutant	Initial concentration	Mechanism of removal	% Removal	Reference
*Ludwigia octovalvis*	Gasoline	2,07,800 mg/kg TPH	Biosurfactant enhanced rhizodegradation	93.5	Almansoory et al. (2015)
*Aegiceras corniculatum*	Brominated diphenyl ethers (BDE-47)	5 μg/gdw	Biostimulated degradation	58.2	Chen et al. (2015)
*Spartina maritima*	As, Cu, Pb, Zn	5–2153 mg/kg	Bioaugmented rhizoaccumulation	19–65	Mesa et al. (2015)
*Arundo donax*	Cd and Zn	78.9 and 66.6 kBq/dm^3^ respectively	Rhizofiltration	100	Dürešová et al. (2014)
*Eichhorina crassipes* (water hyacinth)	Heavy metals (Fe, Zn, Cd, Cu, B, and Cr)	0.02–20 mg/L	Rhizofiltration	99.3	Elias et al. (2014)
*Phragmites australis*	PAHs	229.67 ± 15.56 μg/g	Rhizodegradation	58.47	Gregorio et al. (2014)
*Plectranthus amboinicus*	Pb	5–200 mg/kg	Rhizofiltration	50–100	Ignatius et al. (2014)
*Luffa acutangula*	Anthracene and fluoranthene	50 mg/kg	Phytostimulation^a^	85.9–99.5	Somtrakoon et al. (2014)
*Dracaena reflexa*	Diesel	1–5 wt%	Rhizodegradation	90–98	Dadrasnia and Agamuthu (2013)
*Sparganium* sp.	Polychlorinated biphenyls	6.260 ± 9.3 10^−3^ μg/g	Biostimulated rhizodegradation	91.5	Gregorio et al. (2013)
*Amaranthus paniculatus*	Ni	25–150 μM	Phytoaccumulation	25–60	Iori et al. (2013)
*Rizophora mangle*	TPH	33,215.16 mg/kg	Phytoextraction and phytostimulation	87	Moreira et al. (2013)
*Populusdeltoides x nigra* and *Arabidopsis thaliana*	Silver nanoparticles and Ag^+^	0.01–100 mg/L	Phytoaccumulation	20–70	Wang et al. (2013)
*Carex pendula*	Pb	1.0–10 mg/L	Rhizofiltration		Yadav et al. (2011)

*PAHs* polyaromatic hydrocarbons, *TPH* total petroleum hydrocarbon

^a^Hypothetical, needs further investigation

One of the major advantages of using plants to remediate polluted site is that some precious metals can bioaccumulate in some plants and recovered after remediation, a process known as phytomining. A study by Wu et al. (2015) reported the potential applications (food, feedstuff, biofortification of agricultural products) of Selenium-enriched material recovered from phytoremediation sites. Other advantages of phytoremediation include: low cost, environmentally friendly, large-scale operation, low installation and maintenance cost, conservation of soil structure, prevention of erosion and leaching of metal (Van Aken 2009; Ali et al. 2013). Moreover, following phytoremediation, there might be improved soil fertility due to input of organic matter (Mench et al. 2009). However, longer remediation time, pollutant concentration, toxicity and bioavailability to plant, depth of plant roots and plant slow growth rate are likely to limit the application of phytoremediation (Kuiper et al. 2004; Vangronsveld et al. 2009; Ali et al. 2013). In some cases, harvesting of plant for biomass management following remediation might incur additional cost (Wang et al. 2012a, b). Besides, there is a possibility that accumulated toxic contaminants may be transferred along food chain. Plants by their nature are autotrophic (unable to use organic compounds as sources of carbon and energy), therefore lack catabolic enzymes needed to fully mineralize organic pollutants to carbon dioxide and water; this presents another pitfall for phytoremediation (Lee 2013).

Recombinant deoxyribonucleic acid (DNA) technology has been used to regulate the expression of some plant specific genes in order to increase metabolism and tolerance to heavy metals (Dowling and Doty 2009). Composting of contaminated soil before planting resulted in enhanced TPH degradation, which in turn favoured rhizodegradation by *Suaeda glauca* (Wang et al. 2011). It thus implies that pretreatment and/or amendment of heavily polluted site prior to planting of plants will help improve phytoremediation efficacy by increasing microbial diversity and activity, and at the same time reducing pollutant toxic effects to plants. Recently, Thijs et al. (2016) proposed a competition-driven model for rhizosphere-microbiome interaction, in order to understand and identify factors that play crucial role toward assembly of beneficial (plant-growth promoting (PGP) and degrading) microbiota during phytoremdiation processes. Four major strategies (plant selection in function of microbiome, root exudate interference, disturbance, and feeding of the supply lines) were identified as the strategies to adopt to ensure that in polluted sites, opportunistic and pathogenic microbial populations are kept in check, to enable improved phytoremediation processes by degradative and PGP microbes. Further, it was suggested that plant-microbiome interaction might not always be optimal for phytoremediation; therefore, human interventions are required to optimize such interaction for enhance contaminant removal. More so, addition of organic waste (brewery spent grains) to waste lubricating oil contaminated soil enhanced the growth of *Jatropha curcas* and microbial proliferation at the rhizosphere, resulting in additional 33 % contaminant removal from 2.5 % used lubricating oil contaminated soil compared to treatment with *J. curcas* alone (Agamuthu et al. 2010). Other integrated approaches to enhance phytoremediation in order to make it a reliable and efficient technique, have been described (Wenzel 2009; Schwitzguébel 2015).

## Permeable reactive barrier (PRB)

This technique is mostly perceived as a physical method for remediating contaminated groundwater, due to its design and mechanism of pollutant removal. Nevertheless, researchers (Thiruvenkatachari et al. 2008; Obiri-Nyarko et al. 2014) reported that biological reaction is one of the several mechanisms (degradation, precipitation and sorption) of pollutant removal in PRB technique. Although alternative terms such as biological PRB, passive bioreactive barrier, bio-enhanced PRB have been proposed to accommodate the bioremediation or biotechnology aspect of the technique, the role of microorganisms have been reported to be mostly enhancement rather than an independent biotechnology (Philp and Atlas 2005). In this section, PRB will be used to describe all variants of this technique including the permeable reactive barrier itself unless otherwise stated. In general, PRB is an in situ technique used for remediating groundwater polluted with different types of pollutants including heavy metals and chlorinated compounds (Table 3). In this technique, a permanent or semi-permanent reactive barrier (medium) mostly made up of a zero-valent iron (García et al. 2014; Zhou et al. 2014) is submerged in the trajectory of polluted groundwater. As polluted water flows through the barrier under its natural gradient, pollutants become trapped and undergo series of reactions resulting in clean water in the flow through (Thiruvenkatachari et al. 2008; Obiri-Nyarko et al. 2014). Ideally, the barriers are usually reactive enough to trap pollutants, permeable to allow the flow of water but not pollutants, passive with little energy input, inexpensive, readily available and accessible (De Pourcq et al. 2015). The effectiveness of this technique depends mostly on the type of media used, which is influenced by pollutant type, biogeochemical and hydrogeological conditions, environmental and health influence, mechanical stability, and cost (Obiri-Nyarko et al. 2014; Liu et al. 2015). Recently, researchers have focused on coupling PRB and other methods such as electrokinetics for treatment of different class of pollutants (García et al. 2014; Mena et al. 2015; Ramírez et al. 2015). It was reported that 90 % nitrate removal from spiked clay soil was achieved in 1 week when electrokinetic and PRB techniques were coupled (García et al. 2014). Similarly, Mena et al. (2015) reported 30 % diesel removal from clay soil after 2 weeks of operation, when electrokinetic soil flushing was combined with biological-PRB (Bio-PRB). In addition, Ramírez et al. (2015) reported 39 % reduction in diesel biodegradable fractions after 2 weeks, when Bio-PRB was coupled with electrokinetics for treatment of diesel-polluted soils. Apparently, these combined techniques allowed polluted soil to maintain appropriate environmental conditions (pH, temperature, nutrients) needed for microbial growth, and resulted in surfactant biomass distribution across such polluted soil. Interestingly, a white-rot fungus (*Trametes versicolor*) when used as a bio-barrier brought about 97 % degradation of Orange G dye in an artificial laboratory-scale aquifer, thus demonstrating the potentials of the fungus for use as a barrier (PRB) in natural aquifers (Folch et al. 2013).

**Table 3:** Some pollutants removed by permeable reactive barriers (PRBs) technique

Reactive material	Nature of pollutant	Initial concentration	Mechanism of pollutant removal	% Removal	References
Clay	Cs-137	10^5^ Bq/m^3^	Sorption		De Pourcq et al. (2015)
Oxygen reactive compound and clinoptilolite	NH_4_–N	5–11 mg/L	Ion exchange and biological nitrification	>99	Huang et al. (2015)^a^
Natural pyrite (FeS_2_)	Cr(VI)	10–100 mg/L	Sorption	27–100	Liu et al. (2015)
Zero-valent iron coupled with polyhydroxybutyrate	1, 2-dichloroethane	10 mg/L	Biological degradation	20–80	Baric et al. (2014)
Mixture of zero-valent iron, Zeolite and activated carbon	Landfill leachate			55–94	Zhou et al. (2014)
Bio-barrier (*Arthrobacter viscosus*)	Polyaromatic hydrocarbons	100 μM	Biodegradation	>80	Ferreira et al. (2013)
Bio-barrier (*Trametes versicolor*, white-rot fungi)	Orange G dye	150 mg/L	Biodegradation	97	Folch et al. (2013)
Organic substrates and zero-valent iron (ZVI)	Heavy Metals (Al, Zn and Cu)	15, 20 and 1.2 mg/L	Precipitation	>95	Gibert et al. (2013)
Granular oxygen-capturing materials (ZVI powder, sodium citrate and inorganic salts) and granular activated carbon	Nitrate and nitrite	40 mg/L	Biodegradation	>94	Liu et al. (2013)
Bioaugumented Bio-barrier (*Mycobaterium* sp. and *Pseudomonas* sp. immobilized bead) PRB	Benzene, toluene, ethylbenze and xylene (BTEX)	100 mg/L	Biodegradation	84–97	Xin et al. (2013)
Granular iron	Chlorinated volatile organic compounds (VOC)		Degradation		Vogan et al. (1999)^a^

^a^Pilot-scale study

During performance evaluation of PBR for remediation of dissolved chlorinated solvents in groundwater, formation of carbonate precipitate in the iron zone was found not to be the major limitation to the observed performance; rather, accurate measurement of groundwater velocity through a PRB was implicated (Vogan et al. 1999). Although maintaining barrier reactivity is vital for performance of PRB technique, preserving the barrier permeability is crucial for PRB success and can be achieved by maintaining appropriate particle size distribution (Mumford et al. 2014). Decrease in long-term performance due to reduction in reactivity of the barrier, zero-valent iron (ZVI), loss of porosity and inability to apply the technique to site contaminated with some chlorinated hydrocarbons and recalcitrant compounds are amongst the major operational challenges associated with PRB technique. Nevertheless, it was reported that polyhydroxybutyrate (PHB), a biodegradable polymer, has a slow-release nutrient (carbon) capability, which promoted biological activity when used as a barrier, resulting in enhanced removal of chlorinated compounds (Baric et al. 2014). Variations in climatic conditions, which can cause difficult hydrogeological site characterization, together with design flaws can result in reduced efficiency of PRB (Henderson and Demond 2007). Therefore, cost-effective advanced site characterization methods and improved PRB designs will in turn increase the effectiveness of the technique (Gibert et al. 2013). Furthermore, the use of iron sulphide (FeS) barrier would help overcome some of the challenges (loss of permeability under certain geological conditions) associated with the use of ZVI (Henderson and Demond 2013). In addition, model significant uncertainties are likely to affect the extrapolation of PBR performance based on laboratory-scale column experiments; these uncertainties can be reduced by independent experiments and field observation geared towards better understanding of surface deactivation mechanism in iron PRBs (Carniato et al. 2012). Other designs, reactive media, advantages, limitations and contaminants removed by PRB technique have been extensively described (Thiruvenkatachari et al. 2008; Obiri-Nyarko et al. 2014).

## Intrinsic bioremediation

Intrinsic bioremediation also known as natural attenuation is an in situ bioremediation technique, which involves passive remediation of polluted sites, without any external force (human intervention). The process relies on both microbial aerobic and anaerobic processes to biodegrade polluting substances including those that are recalcitrant. The absence of external force implies that the technique is less expensive compared to other in situ techniques. Nevertheless, the process must be monitored in order to establish that bioremediation is ongoing and sustainable, hence the term, monitored natural attenuation (MNA). Further, MNA is often used to represent a more holistic approach to intrinsic bioremediation. According to the United States National Research Council (US NRC), there are three criteria that must be met in intrinsic bioremediation and these include: demonstration of contaminants loss from contaminated sites, demonstration based on laboratory analyses that microorganisms isolated from contaminated sites have the innate potentials to biodegrade or transform contaminants present at contaminated site from which they were isolated and evidence of realization of biodegradation potentials in the field (Philp and Atlas 2005). In line with these criteria, M’rassi et al. (2015) isolated hydrocarbon-degrading bacteria from refinery oil-contaminated soil, and demonstrated the biodegradation potentials of the isolates by growing them on mineral salt medium with saturated and unsaturated hydrocarbon substrates as sole carbon sources, and also by their capacities to reduce hydrocarbon concentrations. It was further reported that during monitoring of intrinsic bioremediation of chronically polluted marine coastal environment, the most polluted sediments tended to have higher total bacterial diversity, abundance and culturable hydrocarbon degraders and contributed to natural attenuation of such site; therefore, suggesting that bacterial communities could be used as sensitive indicators of contamination in marine sediment (Catania et al. 2015). With respect to chlorinated compounds, Adetutu et al. (2015) compared the effectiveness of three treatments (biostimulation, biostimulation-bioaugmentation, and monitored natural attenuation) towards dechlorination of ground water contaminated with trichloroethene (TCE) and observed successful reduction in TCE concentration below that stipulated by United States Environmental Protection Agency (US EPA). MNA is widely gaining acceptance in most European countries with exception of very few, due to cold climate condition that is likely to exert negative effect on biodegradation process (Declercq et al. 2012). Furthermore, biodegradation has been implicated as the main mechanism of pollutant removal during intrinsic bioremediation (MNA).

One of the major limitations of intrinsic bioremediation is that it might take a longer time to achieve the target level of pollutant concentration, given that no external force is incorporated to expedite the remediation process. It thus follows that prior to application of intrinsic bioremediation, risk assessment needs to be carried out to ensure that remediation time is less than the time stipulated for pollutant to reach exposure point relative to the closest human and animal populations. Moreover, it was reported that intrinsic bioremediation does not result in adequate polyaromatic hydrocarbon (PAH) removal and corresponding reduction in polluted soil eco-toxicity (García-Delgado et al. 2015).

## Bioremediation prospects

It is clear from the foregoing that bioremediation techniques are diverse and have proven effective in restoring sites polluted with different types of pollutants. Microorganisms play crucial role in bioremediation; therefore, their diversity, abundance and community structure in polluted environments provide insight into the fate of any bioremediation technique provided other environmental factors, which can impede microbial activities are maintained at the optimal range. Molecular techniques such as ‘Omics’ (genomics, metabolomics, proteomics and transcriptomics) have contributed towards better understanding of microbial identification, functions, metabolic, and catabolic pathways, in this way overcoming the limitations associated with microbial culture-dependent methods. Nutrient limitation, low population or absence of microbes with degradative capabilities, and pollutant bioavailability are among the major pitfalls, which may hinder the success of bioremediation. Since bioremediation depends on microbial process, there are two major approaches to speed up microbial activities in polluted sites, namely: biostimulation and bioaugmentation. Biostimulation involves the addition of nutrients or substrates to a polluted sample in order to stimulate the activities of autochthonous microbes. As microorganisms are ubiquitous, it is apparent that pollutant degraders are naturally present in polluted sites, their numbers and metabolic activities may increase or decrease in response to pollutant concentration; hence, the use of agro-industrial wastes with appropriate nutrient composition especially nitrogen, phosphorus and potassium, will help solve the challenge of nutrient limitation in most polluted sites. Nonetheless, it was reported that excessive addition of stimulant resulted in suppressed microbial metabolic activity and diversity (Wang et al. 2012b). On the other hand, bioaugmentation is a critical approach aimed at introducing or increasing microbial population with degradative capabilities. Microbial consortium has been reported to degrade pollutants more efficiently than pure isolates (Silva-Castro et al. 2012). This is due to metabolic diversities of individual isolates, which might originate from their isolation source, adaptation process, or as a result of pollutant composition, and will bring about synergistic effects, which may lead to complete and rapid degradation of pollutants when such isolates are mixed together (Bhattacharya et al. 2015). More so, Sun et al. (2012) observed that both bioaugmentation and biostimulation were effective in removing pollutant such as polyaromatic hydrocarbons (PAHs) from heavily polluted sample compared to non-amended setup (control). Nevertheless, biostmulation was observed to be more effective in removing low molecular weight (LMW) PAHs and contributed to higher percentage (33.9 %) of total PAHs removal compared to 26.8 % achieved with bioaugmentation. At the same time, bioaugmentation was observed to be more effective in removing high molecular weight (HMW) PAHs from polluted sample used for the pilot study, resulting in >22 % reduction in HMW–PAHs, whereas with biostimulation, the maximum reduction in individual HWM–PAHs (4–6 ring-PAHs) were only 10.85 %. As expected, when both approaches were combined, higher reduction in both LMW and HMW–PAHs were obtained 43.9 and 55.0 %, respectively. This suggests that removal of HMW–PAHs, which are of public health concern in polluted environment, could be more efficient if microbes with special degradative capabilities are incorporated while stimulating resident microbes with nutrients, rather than relying on a single approach alone. Although bioaugmentation has proven effective, competition between endogenous and exogenous microbial populations, the risk of introducing pathogenic organisms into an environment, and the possibility that the inoculated microorganisms may not survive in the new environment make bioaugmentation a very skeptical approach. The use of agar, agarose, alginate, gelatin, gellan gum and polyurethane as carrier materials will help solve some of the challenges associated with bioagumentation (Tyagi et al. 2011). Furthermore, microbial fuel cells (MFCs) supplemented with inocula (*Shewanella oneidensis* MR1 14063 and *Pseudomonas aeruginosa* NCTC 10662) have been reported as a promising approach for remediation of phenanthrene polluted site (Adelaja et al. 2013). In addition, Fodelianakis et al. (2015) reported that under optimal environmental conditions, indigenous microbes at polluted site would likely degrade pollutant better than allochthonous microbes. In order to improve pollutant availability to degrading microbes, especially in aged and polyaromatic hydrocarbon polluted environment, surfactants are usually used to induce desorption and solubilization of pollutant, thus increasing mass transfer. Biosurfactants are preferred to chemical counterparts due to their environmentally friendly and biodegradable features. However, high production cost and low scalability make large-scale application of biosurfactants to polluted site uneconomical. Incorporation of agro-industrial wastes as nutrient sources for putative biosurfactant producers during fermentation may increase biosurfactant yield.

Simultaneous application of multiple bioremediation techniques during remediation will help increase remediation efficacy (by reducing the weakness of individual technique), and at the same time reduce cost (Cassidy et al. 2015; García-Delgado et al. 2015; Martínez-Pascual et al. 2015). Application of combined metrics of spatial configuration of bacterial dispersal networks will be a good indicator of biodegradation performance (Banitz et al. 2016). Enhancing bioremediation efficacy with controlled use of genetically engineered microorganisms (GEM) is a promising approach. This is due to possibility of engineering a designer biocatalyst (GEM, which can effectively degrade a target pollutant including recalcitrant compounds) by incorporating a novel and efficient metabolic pathways, widening the substrate range of existing pathways and increasing stability of catabolic activity (Paul et al. 2005). Nevertheless, horizontal gene transfer and uncontrolled multiplication of GEM in an environment limit the application of such a promising approach. Notwithstanding, bacterial containment systems, in which any GEM escaping an environment will be killed by induction of controlled suicide systems will help gain public acceptance of using GEM to restore polluted environment. Further, engineering microorganisms with degradative pathway of a target compound using synthetic biology approach could improve bioremediation efficiency. The use of nanomaterials could help reduce the toxicity of pollutant to microorganisms. Nanomaterials increase surface area and lower activation energy, thereby increasing the efficiency of microorganisms in degradation of waste and toxic materials, resulting in overall reduction in remediation time and cost (Rizwan et al. 2014).

## Conclusion

The foremost step to a successful bioremediation is site characterization, which helps establish the most suitable and feasible bioremediation technique (ex situ or in situ). Ex situ bioremediation techniques tend to be more expensive due to additional costs attributed to excavation and transportation. Nonetheless, they can be used to treat wide range of pollutants in a controlled manner. In contrast, in situ techniques have no additional cost attributed to excavation; however, cost of on-site installation of equipment, coupled with inability to effectively visualize and control the subsurface of polluted site may render some in situ bioremediation techniques inefficient. Consequently, cost of remediation apparently is not the major factor that should determine the bioremediation technique to be applied to any polluted site. Geological characteristics of polluted site(s) including soil type, pollutant depth and type, site location relative to human habitation and performance characteristics of each bioremediation technique should be incorporated in deciding the most suitable and efficient method to effectively treat polluted sites.
